# Advances in Nano-Drug Delivery Systems for Chronic Autoimmune Diseases: A Focus on Diabetes Mellitus, Inflammatory Bowel Disease, and Rheumatoid Arthritis

**DOI:** 10.3390/molecules31122094

**Published:** 2026-06-14

**Authors:** Mengqing Hu, Yimiao Zhou, Lin Yang, Liquan Zhou, Xiao Liu, Tianjin Ma, Zuowei Xiao

**Affiliations:** 1 Hunan Engineering and Technology Research Center for Health Products and Life Science, School of Pharmacy, Hunan University of Chinese Medicine, Changsha 410208, China; 20243773@stu.hnucm.edu.cn (M.H.); lauriechow@126.com (Y.Z.); zlq8067@126.com (L.Z.); 2 Xiangxing College, Hunan University of Chinese Medicine, Xiangyin Campus, Yueyang 414615, China; 3 Homologous Innovation Laboratory of Medicine and Food, Hunan University of Chinese Medicine, Changsha 410208, China; 4 Guizhou Institute of Crop Germplasm Resources, Guizhou Academy of Agricultural Sciences, Guiyang 550006, China; yanglin070809@163.com (L.Y.); 15761635227@163.com (X.L.); mtj372613899@126.com (T.M.)

**Keywords:** autoimmune diseases, nanocarriers, drug delivery, diabetes mellitus, inflammatory bowel disease, rheumatoid arthritis

## Abstract

The global prevalence of autoimmune diseases ranges from 3% to 8%, with women at a significantly higher risk than men. The core mechanisms underlying these diseases include impaired T-cell and B-cell immune tolerance, abnormal cytokine production, and aberrant activation of related signaling pathways. Conventional treatments primarily focus on suppressing immune responses, but their efficacy remains limited and they are often associated with substantial side effects. Nanomedicine leverages nanoscale materials to enable precise diagnosis and targeted therapy. Nanocarriers can penetrate biological barriers, enhance cellular uptake, and prolong circulation time in vivo, demonstrating considerable potential for drug delivery. Common nanoscale drug delivery platforms include nanoparticles, polymeric micelles, liposomes, dendrimers, mesoporous materials, hydrogels, and exosomes. Each carrier type possesses distinct characteristics in terms of drug-loading capacity, stability, responsiveness, and biocompatibility, thereby enabling targeted delivery and controlled release. This review summarizes recent advances in nano-delivery technologies for three representative chronic autoimmune diseases: diabetes mellitus (DM), inflammatory bowel disease (IBD), and rheumatoid arthritis (RA). Nano-delivery systems can improve therapeutic outcomes by optimizing drug delivery, targeting complications, and modulating the pathological microenvironment. They enhance drug bioavailability, reduce off-target and systemic adverse effects, and provide novel strategies for the precise and efficient treatment of chronic autoimmune diseases.

## 1. Introduction

Autoimmune diseases are a group of conditions in which the body’s immune system mistakenly attacks its own cells and tissues. This abnormal response triggers chronic inflammation and causes damage to multiple organs and tissues, leading to various clinical symptoms and complications. Globally, the overall prevalence of autoimmune diseases ranges from 3% to 8%, with women accounting for 78% to 85% of patients [1]. In the United States, over 15 million people have been diagnosed with at least one autoimmune disease, with 34% having multiple conditions; women are approximately twice as likely to develop these diseases as men [2]. Currently, more than 80 types of autoimmune diseases have been identified, and these diseases exhibit significant differences in target organs, immunopathological mechanisms, and clinical progression [3]. Autoimmune diseases can be classified into organ-specific autoimmune diseases and systemic autoimmune diseases. DM, IBD, multiple sclerosis, psoriasis, and Hashimoto’s thyroiditis all fall under the category of organ-specific autoimmune diseases, in which the patient’s immune system targets specific organs or tissues. In contrast, systemic autoimmune diseases are characterized by the immune system targeting autoantigens that are present in virtually all types of cells, such as in RA and systemic lupus erythematosus [4].

The pathogenesis of autoimmune diseases is characterized by complexity. These diseases share common pathogenic mechanisms, primarily manifested by the disruption of immune tolerance in T cells and B cells, accompanied by abnormal cytokine production and the abnormal activation of related signaling pathways [5]. Due to the highly complex structure and function of the immune system itself, the progression of autoimmune diseases is often unpredictable. Current treatment strategies primarily focus on suppressing immune responses and controlling clinical symptoms; however, these approaches remain limited in achieving long-term, stable disease management and are often associated with significant side effects [6]. Therefore, it is particularly crucial to thoroughly investigate the specific mechanisms driving autoimmune responses. Through the use of nanomaterials, effective drug delivery devices can be developed. In addition, innovative drug targets may help restore immune tolerance or selectively control pathogenic immune signaling. Together, these advances are expected to enable more focused, efficient, and safe treatment strategies for autoimmune diseases and may create new opportunities for treating chronic autoimmune diseases in a targeted manner.

Nanomedicine is a new discipline that uses nanoscale materials to provide assistance in disease diagnosis and therapy. As an example, the methods and materials created based on nanotechnology have already been used in the diagnosis and treatment of cancer [7].The interdisciplinary integration of nanotechnology and pharmacy holds great potential for development and has made rapid progress in recent years. Dendrimers, micelles, solid lipid nanoparticles (SLNs), carbon nanotubes, liposomes, and exosomes are common nanocarriers. They are composed of organic or inorganic compounds, polymers, or metals and are frequently used in targeted drug delivery and controlled-release systems [8,9]. However, the size, charge, morphological structure, and compositional makeup of nanoparticles can all influence their in vivo distribution, cellular penetration efficiency, and the release rate of the loaded drug [10]. Compared to micron-sized particles, nanocarriers possess significant advantages, as their minute size enables them to overcome biological barriers, enhance cellular uptake, and prolong circulation time. Unlike micrometer-sized particles, nanoparticles can achieve passive tumor targeting through enhanced permeation and retention effects and penetrate tissues more deeply [11]. Therefore, under ideal conditions, nanoparticles and materials with good biodegradability [12] and biocompatibility [13] are excellent choices for drug delivery in the biomedical field.

Given the immense potential of nanotechnology in the field of human disease intervention, this review aims to systematically summarize various drug delivery strategies mediated by nanomaterials. It focuses on recent advances in their application for the treatment of chronic autoimmune diseases, with particular emphasis on DM, IBD, and RA, and provides an in-depth analysis of the key issues and core challenges currently facing this field.

## 2. Applications of Various Nanocarriers in Three Representative Chronic Autoimmune Diseases: DM, IBD, and RA

**Figure 1:**
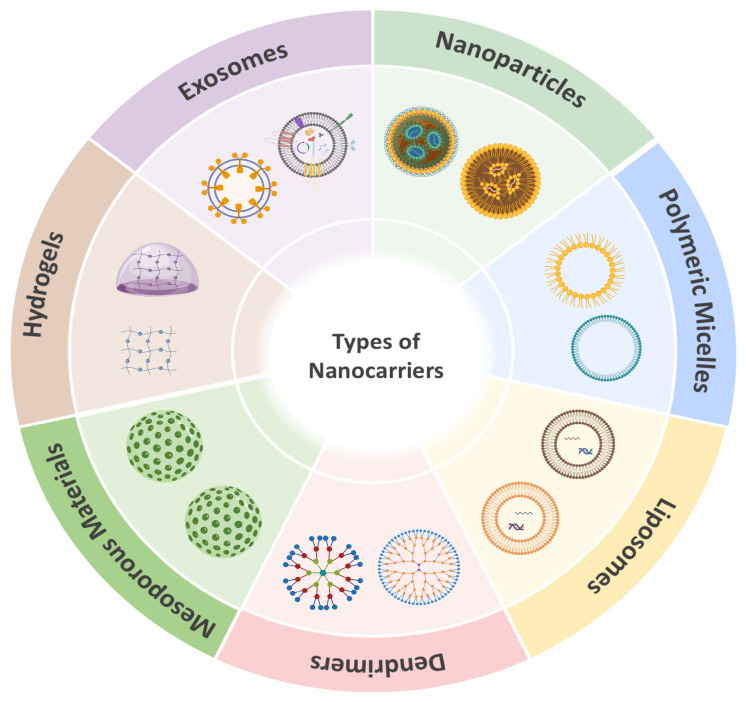
Summary of nano-drug delivery systems for three representative chronic autoimmune diseases: DM, IBD, and RA. Created with BioGDP.com [22].

In the field of disease diagnosis, nanomaterials can leverage high-resolution imaging and highly sensitive sensing technologies to accurately identify various biomarkers, thereby improving the accuracy of early-stage disease screening [14]. In disease treatment, the application of nanoparticles enables targeted drug delivery, which reduces adverse reactions while further enhancing overall therapeutic efficacy [15]. Continuous advancements in controlled-release platforms and nanocarrier technologies are accelerating the development of treatments for chronic autoimmune diseases toward greater precision and efficiency. Nanocarriers serve as highly efficient drug delivery systems capable of loading both natural and synthetic biomolecules, as well as nucleic acid-based drugs, to achieve controlled drug release and exert synergistic effects within tissues, thereby enhancing the efficacy of chemotherapeutic agents. Additionally, various biopolymers can be used to modify their surface and interfacial properties, optimizing delivery performance [16]. Nanomaterials with different structures and morphologies are combined with drug molecules to construct nanoscale drug delivery systems. Based on recent technological advancements, the most widely used drug delivery carriers currently include nanoparticles [17], liposomes [18], polymeric micelles [19], and exosomes [20]. The variety of materials used for drug delivery is rapidly expanding, and these materials demonstrate significant potential for application in both disease diagnosis and treatment [21]. Details of various nano-drug delivery systems are shown in Figure 1.

**Figure 2:**
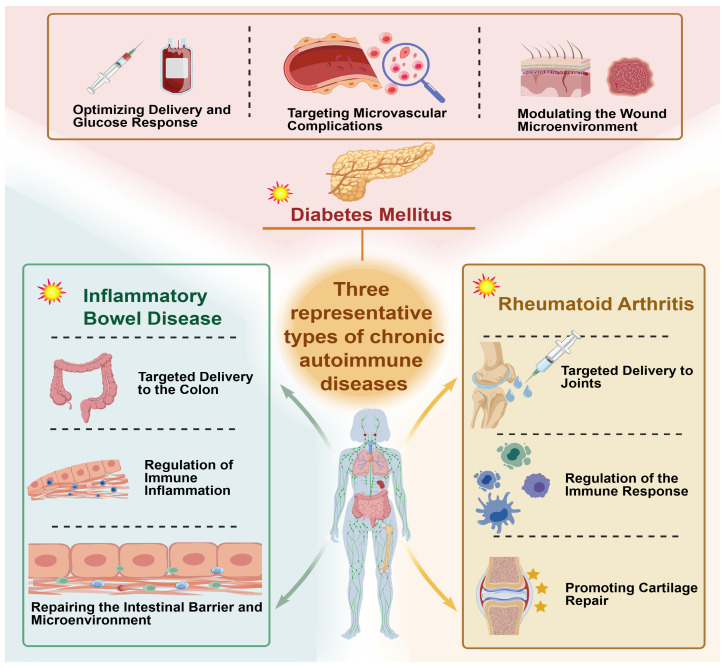
Three representative types of chronic autoimmune diseases and treatment strategies for each. Created with BioGDP.com [22].

The following discussion will focus on three chronic autoimmune diseases—DM, IBD, and RA—addressing core pathological mechanisms such as damage to pancreatic β-cells, chronic intestinal inflammation, and synovial infiltration. Therapeutic strategies for three chronic autoimmune diseases are shown in Figure 2. The targeted modification, smart response, and non-invasive delivery capabilities of nanotechnology offer new strategies for overcoming therapeutic bottlenecks in the treatment of these diseases.

### 2.1. Applications of Nano-Drug Delivery Systems in DM 

DM is a chronic metabolic disorder caused by insufficient insulin secretion or impaired insulin action, characterized primarily by persistent hyperglycemia. In 2024, approximately 3.4 million people worldwide died from diabetes, and it is projected that by 2045, the global adult population with DM will reach 783 million, with the disease burden continuing to increase [23]. Based on pathological and physiological mechanisms, DM is primarily classified into type 1 and type 2 diabetes. Type 1 diabetes results from an absolute deficiency of insulin due to autoimmune-mediated destruction of β-cells, while type 2 diabetes is characterized by insulin resistance and progressive β-cell dysfunction. Additionally, the spectrum includes gestational diabetes and other specific types [23,24]. If diabetes is poorly controlled over the long term, it can lead to various secondary complications, such as diabetic nephropathy, diabetic retinopathy(DR), diabetic peripheral neuropathy, coronary artery disease, cerebrovascular disease, and peripheral artery disease [25]. Current standard treatment for diabetes is based on lifestyle interventions combined with oral hypoglycemic agents such as metformin, sulfonylureas, SGLT-2 inhibitors, and DPP-4 inhibitors, as well as insulin injections. Although these approaches can effectively control blood glucose levels, they still have limitations, including low drug bioavailability, the need for frequent dosing, poor patient compliance, and adverse reactions associated with long-term use [25,26]. In contrast, nanodelivery systems, by encapsulating drugs within carriers such as liposomes, polymeric nanoparticles, or nanovesicles, protect the drugs from degradation by the acidic environment and enzymes of the gastrointestinal tract, enhance intestinal mucosal permeability, and enable targeted delivery and controlled release. This significantly improves oral bioavailability, offering a new strategy for diabetes management that is both more precise and highly effective [27,28,29]. Applications of specific nano-drug delivery systems in DM are summarized in Table 1.

#### 2.1.1. Optimizing Delivery and Glucose Response

Targeted drug delivery is a rapidly evolving method of drug administration that utilizes carrier systems to transport active substances to target organs or cells. This enables therapeutic drugs to accumulate precisely at the site of the lesion, increasing local drug concentrations and effectively reducing side effects [30]. Targeted delivery systems consist of three core elements: the drug component, the delivery carrier, and the target cells, tissues, or organs to be treated. The carrier itself is non-toxic, biodegradable, low in immunogenicity, and easily detectable. It is used to encapsulate drug molecules and deliver them to the target organ, while simultaneously enhancing drug permeability and bioavailability [31]. Nanoparticles can precisely deliver drugs to target cells; their delivery efficiency is far higher than that of micron-sized particles, and they are less likely to be rapidly cleared by the immune system. Polymer nanoparticles are colloidal systems composed of natural or synthetic polymers, primarily prepared through two methods: direct polymerization of monomers or the dispersion of preformed polymers [32]. Polymer nanoparticles allow for precise control over physicochemical properties such as particle size, shape, structure, and charge, enabling targeted delivery. They can also encapsulate various types of therapeutic agents and achieve efficient drug release in response to external or internal stimuli. Therefore, the design of efficient polymer nanoparticles for drug delivery is of critical importance [33]. Studies have shown that ANG nanoparticles can upregulate LRP-1 expression on the apical surface of Caco-2 cells and promote its redistribution to the basolateral side, thereby establishing a transcellular transport pathway from the apical to the basolateral side. Thanks to this enhanced transcytosis, insulin-loaded ANG nanoparticles demonstrated higher absorption efficiency in diabetic rats, resulting in a maximum reduction in blood glucose levels of 61.46% [34]. In addition to enhancing nanoparticle trans-epithelial transport through ligand modification, the development of smart drug delivery systems using materials responsive to the pathological microenvironment is also a key strategy for optimizing diabetes treatment. Volpatti et al. [35] designed a glucose-responsive nanoparticle based on the reversible binding of glucose to a phenylboronic acid derivative, enabling rapid insulin release upon elevated blood glucose levels and cessation of release once blood glucose returns to normal. A single injection of this formulation can maintain blood glucose within the normal range for up to 12 hours while preventing hypoglycemic events. Compared to injectable smart drug delivery systems, oral insulin formulations offer higher patient compliance but must overcome enzymatic, chemical, and epithelial barriers in the gastrointestinal tract. Hydrogels are a class of three-dimensional cross-linked supramolecular networks with high water-absorption capacity and are among the biomaterials with the greatest potential for development [36]. Hydrogels can be prepared through various methods. For example, the overall gel structure can be formed through several cross-linking mechanisms. These include covalent bonds, ionic bonds, physical entanglement, hydrogen bonds, and other interactions between polar molecules [37]. A pH-responsive microalgal hydrogel-based oral insulin delivery system derived from Chlorella (CV@INS@ALG), cross-linked with calcium alginate, effectively protects insulin from degradation by gastric acid and enzymes and triggers drug release under intestinal pH conditions. CV@INS@ALG demonstrated a more sustained and effective hypoglycemic effect than subcutaneous insulin injection, with no intestinal toxicity [38].

#### 2.1.2. Targeting Microvascular Complications

Diabetic pan-vascular disease is a clinical syndrome characterized by vascular lesions in multiple sites throughout the body, primarily manifested by the coexistence of atherosclerosis in large vessels and structural and functional abnormalities in microvessels. Large-vessel lesions and microvascular damage often coexist in the heart, brain, and peripheral tissues, whereas the kidneys and retina are primarily affected by microvascular lesions [39]. Microvascular complications are the primary cause of disability and mortality in diabetes, and targeted delivery systems can concentrate therapeutic drugs at the sites of lesions. Early oxidative stress plays a critical role in the progression of DR. Li et al. [40] designed a reactive oxygen species (ROS)-responsive nanodelivery system loaded with essential oil of Azalea, which significantly inhibits abnormal cell proliferation and reduces oxidative stress and inflammatory responses. This system not only alleviates early pathological changes in the retina but also mitigates multi-organ damage. Diabetic nephropathy is also one of the primary manifestations of microvascular complications, and renal interstitial fibrosis is a key pathological process in its progression to end-stage renal disease. Liposomes are spherical vesicles composed of one or more concentric phospholipid bilayers enclosing an internal aqueous core [41]. Their internal aqueous phase can be used to encapsulate hydrophilic drugs, while the phospholipid bilayers are suitable for loading lipophilic drugs. Liposomes possess nanoscale particle sizes, structural characteristics similar to biological membranes, and excellent biocompatibility. Consequently, they are increasingly recognized as carrier systems in the field of drug delivery [42]. Liposomes can achieve passive accumulation in pathological tissues through the enhanced permeability and retention effect. Compared to unencapsulated free drugs, this passive targeting strategy can significantly improve pharmacokinetic behavior, reduce non-specific toxicity, and widen the therapeutic window [43]. Carthamin yellow (CY)-loaded glycyrrhetinic acid (GA) liposomes can improve renal function, reduce collagen deposition and the area of fibrosis, and downregulate TGF-β1 expression, with therapeutic efficacy superior to that of vitamin E or free CY. By inhibiting the activation of the TGFBR1/Smad2/Smad3 signaling pathway, these liposomes effectively suppress TGF-β1-stimulated proliferation of human renal interstitial fibroblasts and reduce the expression of fibrosis markers such as fibronectin(FN) and type I collagen [44]. In addition to liposomal delivery, Mesoporous materials have become ideal carriers for drug delivery and release due to their excellent biocompatibility and high thermodynamic stability [45]. Mesoporous silica nanoparticles (MSNs) possess characteristics such as a large specific surface area, tunable mesoporous structures, substantial pore volume and pore size, and ease of surface functionalization. They can efficiently load therapeutic drugs such as genes, peptides, and proteins through chemical bonding or electrostatic adsorption, enabling precise targeted drug delivery [46,47]. One such example is a FN-coated, thiol-linked methoxypolyethylene glycol thiol-modified mesoporous silica nanoparticle (FN@ EGCG-MSN-TK NPs). Under the trigger of high levels of ROS in an inflammatory environment, FN@EGCG-MSN-TK NPs enable the controlled release of EGCG and exhibit excellent biocompatibility and ROS scavenging capacity. By alleviating oxidative stress, inhibiting apoptosis, enhancing autophagy, and regulating macrophage polarization, these nanoparticles significantly improved pathological damage in diabetic nephropathy [48].

#### 2.1.3. Modulating the Wound Microenvironment

Diabetic wounds represent one of the most challenging public health issues today. Their development is associated with infection, insufficient blood supply, and oxidative stress imbalance, and there is a lack of effective treatment options. Due to their small particle size, high safety profile, and good biocompatibility, nanoparticles possess anti-inflammatory, antibacterial, and antioxidant properties. They can carry various bioactive substances and be combined with biomaterials, demonstrating promising applications in the repair of diabetic wounds [49]. Multifunctional hydrogels, by integrating multiple active ingredients with controlled-release systems, provide a more comprehensive platform for the treatment of diabetic wounds. Dihydromyricetin (DMY) was encapsulated in aldehyde-modified Pluronic F127 micelles, which were then cross-linked with amine-rich polyethyleneimine (PEI) via a Schiff base reaction to construct a multifunctional hydrogel (DPFI). The DPFI hydrogel exerts synergistic effects through the antimicrobial action of PEI and the antioxidant and anti-inflammatory activities of DMY. Concurrently, DMY effectively scavenges ROS, induces the expression of antioxidant enzymes, and promotes the conversion of M1 macrophages to M2 macrophages, thereby alleviating inflammation. Notably, the DPFI hydrogel also exhibits intrinsic blood glucose-regulating functions, which help reduce hyperglycemia-related wound complications [50]. A hyperglycemic environment also poses a serious threat to the survival of multi-vessel flaps during surgery, with distal necrosis closely associated with insufficient perfusion in the “choke zone.” By encapsulating metformin-loaded MSNs within exosomes derived from bone marrow mesenchymal stem cells, engineered exosomes (M-MS@EXO NPs) were constructed to achieve targeted delivery of metformin. By inhibiting the TNF/apoptosis signaling pathway and enhancing the VEGF signaling pathway, M-MS@EXO NPs exert dual anti-inflammatory and pro-angiogenic effects, effectively alleviating inflammation in the “choke zone” and promoting neovascularization, thereby reducing distal flap necrosis and significantly improving flap survival rates. Compared with oral metformin, this engineered exosome delivery system enables precise drug delivery within the diabetic microenvironment, offering a new strategy for improving the prognosis of skin flap transplantation in diabetic patients [51].

**Table 1:** Applications of nano-drug delivery systems in DM management.

Therapeutic Strategies	Types of Nanocarriers	Surface Finishing	Drug-Loaded	Animal Models	Function	References
Optimizing Delivery and Glucose Response	Nanoparticle	ANG	INS	STZ, DM rats	LRP1-mediated transport ↑, BG ↓	[34]
	Nanoparticle	n/a	INS + GOx + CAT	STZ, DM mice	Glucose-responsive release, BG ↓	[35]
	Nanoparticle	n/a	MET	STZ, DM rats	Sustained release, BG ↓, islet repair ↑	[52]
	Liposome	n/a	MET	STZ, DM mice	Liver mitochondrial targeting, glucose catabolism ↑, BG ↓	[53]
	Dendrimer	Glucose-binding motif	MitoPBN	STZ, DM mice, DM pigs	Glucose-responsive release, BG ↓	[54]
	Mesoporous material	Amino	Modified INS	HFD, IR mice	Co-delivery, hepatic IR and BG ↓	[55]
	Mesoporous material	FcBP	pFGF21 + Lira	STZ, DM mice	Pro-inflammatory cytokines ↓, macrophage polarization balance ↑, hyperglycemia ↓	[56]
	Exosome	n/a	Exe	STZ, DM mice	Fibroblast proliferation/collagen deposition/fibrosis ↓	[57]
Targeting microvascular complications	Liposome	GA	CY	DN rats	ROS-responsive release, autophagy ↑,M1 polarization ↓	[44]
	Mesoporous material	FN	EGCG	STZ, DM mice	Renal targeting, NF-κB/NLRP3 ↓, renal function ↑	[48]
	Liposome	CS	TAX	STZ, DM mice	ROS ↓,Akt signaling and cell migration ↑	[58]
Regulating the wound microenvironment	Hydrogel	PF127/PEI	DMY	STZ, DM mice	Inflammation ↓,angiogenesis/flap survival ↑	[50]
	Exosome	BMSC-EXO	MET@MSNs	STZ, DM rats	Inflammation/bacteria ↓, angiogenesis ↑	[51]
	Nanoparticle	HA	ORI micelles + Cu(II)-PDA NPs	STZ, DM mice	Pro-inflammatory cytokines ↓, macrophage polarization balance ↑, hyperglycemia ↓	[59]
	Hydrogel	GelMA/DA	MSC-EXOs + MC-EXOs	STZ, DM mice	Inflammation/oxidative stress ↓, angiogenesis ↑, macrophage polarization balance ↑	[60]
	Hydrogel	Fc/CD	BBR-micelles	STZ, DM mice	M2 polarization ↑, inflammation ↓	[61]
	Hydrogel	GelMA/DAS	Lemon-EXOs	STZ, DM rats	Macrophage reprogramming/fibroblast proliferation ↑, sustained release	[62]
	Hydrogel	F127/Gel-BA	CUR-FCHO + Mg micromotors	STZ, DM mice	pH-responsive release, inflammation ↓, angiogenesis/collagen deposition ↑	[63]
	Hydrogel	QCS-BA/KGM	Sanqi EVs	STZ, DM mice	pH-responsive release, angiogenesis/collagen deposition ↑	[64]

Table notes: n/a (not applicable or not available), ↑ (up-regulated, increase, activate), ↓ (down-regulated, decrease, inhibit), ANG (angiopep-2), BBR (berberine), BG (blood glucose), BMSC (bone marrow mesenchymal stem cell), CAT (catalase), CP (choline phosphat), CS (chitosan), CUR (curcumin), CY (carthamin yellow), DAS (dialdehyde starch), DN (diabetic nephropathy), EGCG (epigallocatechin gallate), EVs (extracellular vesicles), EXOs (exosomes), Exe (exenatide), FCHO (aldehyde-functionalized Pluronic F127), Fc/CD (ferrocene/cyclodextrin), FcBP (FcRn-binding peptide), FN (fibronectin), GA (glycyrrhetinic acid), Gel-BA (gelatin-benzaldehyde), GelMA (gelatin methacryloyl), GOx (glucose oxidase), HA (hyaluronic acid), HFD (high-fat diet), INS (insulin), IR (insulin resistance), KGM (konjac glucomannan), Lira (liraglutide), MC (Momordica charantia (bitter melon)), MET (metformin hydrochloride), MSC (mesenchymal stem cell), MSNs (mesoporous silica nanoparticles), PEI (polyethylenimine), PF127 (Pluronic F127), pFGF21 (fibroblast growth factor 21 plasmid), QCS-BA (quaternized chitosan-boronic acid), ROS (reactive oxygen species), STZ (streptozotocin), T1DM (type 1 diabetes mellitus), TAX (taxifolin).

### 2.2. Controlling IBD Using Nanocarriers 

Inflammatory bowel disease (IBD) includes Crohn’s disease (CD) and ulcerative colitis (UC). It is a chronic inflammatory condition of the gastrointestinal tract. Its development is characterized by complex interactions among genetic susceptibility, immune dysregulation, alterations in the gut microbiota, and environmental exposures [65,66]. The epidemiological evolution of IBD is divided into four phases: the emergence phase, the accelerated incidence phase, the compound growth phase of prevalence, and the equilibrium phase of prevalence. In 2020, developing countries were in the emergence phase, newly industrialized countries were in the accelerated phase, and Western countries were in the compound growth phase. In the future, due to the aging of the patient population and the impact of mortality during the COVID-19 pandemic, Western countries will enter the equilibrium phase, where the growth in prevalence stabilizes [67]. Current IBD medications, such as 5-aminosalicylic acid derivatives, corticosteroids, immunosuppressants, and other biologics, all carry risks of inducing immunosuppression and long-term systemic exposure [68]. Compared to traditional methods, nanocarriers offer numerous advantages in IBD treatment, such as the ability to achieve specific localization and targeted delivery of drugs. They can preferentially accumulate in inflamed areas and release the required drug dose at the target site, thereby reducing potential adverse reactions while enhancing the therapeutic efficacy of the treatment. Furthermore, because nanocarriers remain in the inflamed area for a longer duration, their sustained-release properties can further extend the duration of drug action [69]. By enabling colon-targeted delivery, modulating immune and inflammatory responses, and repairing the intestinal barrier and microenvironment, nanodelivery systems offer new strategies for the treatment of IBD. Applications of specific nano-drug delivery systems in IBD are summarized in Table 2.

#### 2.2.1. Targeted Delivery to the Colon

Oral administration targeting the colon is a highly attractive strategy for treating IBD. However, achieving effective drug delivery to the colon remains challenging, necessitating the design of more advanced delivery systems. Nanocarriers have garnered widespread attention as colon-targeted delivery platforms because their small size and structural composition facilitate drug accumulation at the site of action, thereby aiding in localized therapy [70]. Wang et al. [71] prepared curcumin-loaded anionic liposomes (CUR-LPs) with a particle size of approximately 167 nm and a zeta potential of −34 mV, which exhibited good stability in simulated gastric fluid. CUR-LPs significantly alleviated clinical symptoms such as weight loss, diarrhea, and bloody stools, prevented colonic tissue damage and colonic shortening, and reduced levels of inflammatory markers including malondialdehyde, myeloperoxidase, interleukin-6, and tumor necrosis factor-α. Therefore, liposomes show great potential as colon-specific delivery carriers for enhancing the stability and anti-inflammatory effects of curcumin. Silica nanoparticles (5-ASA-SiO_2_ NPs) loaded with 5-ASA were prepared using the microemulsion method and were able to selectively deliver the drug to the inflamed colon in a DSS-induced UC mouse model. Compared with free 5-ASA, these nanoparticles significantly improved the disease activity index and histopathological scores, reduced levels of myeloperoxidase, serum IL-6, and TNF-α, as well as their mRNA expression in the colonic mucosa, thereby effectively enhancing the therapeutic efficacy of UC treatment [72]. Rectal administration allows the drug to act directly on the affected colonic mucosa by avoiding absorption in the upper gastrointestinal tract and first-pass metabolism. However, the efficacy of rectal formulations is often limited by insufficient drug retention time. Mucus-adhering hydrogel-based rectal delivery systems offer a more effective and safer administration strategy for UC treatment. Xu et al. [73] prepared a mucus-adhesive hydrogel (SSZ/Cat-CS) loaded with sulfasalazine (SSZ) using quinidine-crosslinked catechol-modified chitosan (Cat-CS) for rectal administration in the treatment of UC. Compared with oral SSZ, rectal administration of the SSZ/Cat-CS hydrogel demonstrated better therapeutic effects while significantly reducing the plasma concentration of the toxic byproduct sulfonamidopyridine.

#### 2.2.2. Regulation of Immune Inflammation

IBD is an incurable disease characterized by inflammatory immune cell infiltration and inflammatory damage to the intestinal epithelium [74]. To address the issues of instability and poor targeting of oral drugs in the gastrointestinal tract, Zhang et al. [75] employed a one-step surface functionalization technique to prepare PLGA/PLA-PEG-folic acid nanoparticles (NPs-PEG-FA/6-shogaol) loaded with the ginger-derived compound 6-shogaol. NPs-PEG-FA/6-shogaol exhibited good biocompatibility both in vitro and in vivo and were efficiently taken up by C26 cells and activated RAW 264.7 macrophages via receptor-mediated endocytosis. At the same time, these nanoparticles can regulate the expression levels of pro-inflammatory factors (TNF-α, IL-6, IL-1β, iNOS) and anti-inflammatory factors (Nrf-2, HO-1), accelerating colonic wound repair. The shift from synthetic nanoparticles to natural, edible plant-derived liposomes offers a new strategy for the treatment of UC. Ginger-derived lipid vesicles (GDLVs) loaded with CD98-targeting siRNA (siRNA-CD98/GDLVs) demonstrated efficient targeting of colonic tissue following oral administration, significantly reducing CD98 expression levels. GDLVs exhibit good biocompatibility and transfection efficiency, effectively avoiding the potential side effects and non-specific issues associated with traditional synthetic nanoparticles [76]. Exosomes are a class of nanoscale extracellular vesicles, typically 30–200 nm in size [77]. They are enclosed by a lipid bilayer membrane, and their formation begins with budding from the endosomal membrane [78]. This process forms multivesicular bodies, which fuse with the plasma membrane to release exosomes into the extracellular environment. Through interactions with neighboring or distant cells, exosomes play a crucial role in regulating various cellular functions [79]. Exosomes possess inherent low immunogenicity, good biocompatibility, and the ability to carry endogenous bioactive molecules (such as proteins and nucleic acids), demonstrating unique advantages in regulating complex immune-inflammatory networks. Treatment of IBD mice with human umbilical cord mesenchymal stem cell-derived exosomes (hucMSC-Ex) administered via the tail vein demonstrated that hucMSC-Ex effectively suppresses inflammatory responses in vivo and in vitro. The mechanism involves upregulating the expression of SIRT1 and FXR in macrophages, reducing FXR acetylation levels, and thereby inhibiting the activation of the NLRP3 inflammasome, thus blocking the inflammatory process. By targeting macrophages or inhibiting pro-inflammatory pathways, these nanosystems remodel the intestinal immune microenvironment [80].

#### 2.2.3. Repairing the Intestinal Barrier and Microenvironment

The pathogenesis of IBD is associated with an imbalance in the gut microbiome, impaired intestinal barrier function, and dysregulation of the mucosal immune response to gut commensal bacteria. Traditional medical interventions for IBD primarily aim to control symptoms by suppressing the immune response. However, these approaches typically fail to address the underlying causes of IBD. To address this unmet clinical need, nanomedicines capable of targeting inflamed colonic epithelium, modulating the gut microbiome, and promoting local anti-inflammatory immune responses have demonstrated superior efficacy compared to conventional IBD therapies [81]. Tang et al. [82] developed a supramolecular hydrogel platform based on furanaldehyde-functionalized chitosan-mannan polymers and 3 -maleimide-functionalized HP-β-CD, encapsulating kaempferol (Kae) within the hydrophobic cavity while simultaneously incorporating rhubarb-derived nanovesicles (RNs) into the cross-linked network of the hydrogel, thereby forming the Kae/CMCHD@RNs system. RNs and Kae exhibit synergistic effects in treating UC, including reducing inflammation, alleviating oxidative stress, and restoring intestinal barrier function. The Kae/CMCHD@RNs system utilizes pH/enzyme sensitivity to achieve sustained release at the colonic site and enables targeted delivery to macrophages. Exosome-like nanoparticles isolated from edible Portulaca oleracea L(PELNs) effectively inhibit the expression of pro-inflammatory factors (TNF-α, IL-6, IL-12, IL-1β) and myeloperoxidase, while elevating levels of the anti-inflammatory factor IL-10. Furthermore, PELNs maintain the diversity and balance of the gut microbiota and promote the growth of *Lactobacillus reuteri*. By modulating the microbiota-immune axis to treat UC, PELNs offer a new direction for the development of oral, colon-targeted natural nanomedicines [83]. Similar to PELNs, nanovesicles derived from honeysuckle (HNVs) also demonstrate potential for modulating the gut microbiome. Wang et al. [84] isolated and prepared HNVs from honeysuckle. Studies indicate that the therapeutic effects of HNVs are closely associated with beneficial changes in the gut microbiota, including increased abundance of beneficial bacteria, reduced numbers of pathogenic bacteria, elevated levels of short-chain fatty acids, and regulation of bile acid metabolism, thereby maintaining intestinal immune homeostasis.

**Table 2:** Applications of nano-drug delivery systems in the treatment of IBD.

Therapeutic Strategies	Types of Nanocarriers	Surface Finishing	Drug-Loaded	Animal Models	Function	References
Targeted delivery to the colon	Liposome	n/a	CUR	DSS, UC mice	Colon targeting, oxidative stress ↓, inflammation ↓	[71]
	Nanoparticle	n/a	5-ASA	DSS, UC mice	Inflamed-colon targeting, inflammation ↓, histopathology ↑	[72]
	Hydrogel	Cat-CS	SSZ	UC mice	Rectal delivery, colon retention ↑, systemic toxicity ↓	[73]
	Mesoporous material	n/a	MDC	DSS, UC mice	Colon targeting, inflammation ↓, oxidative stress ↓, microbiota homeostasis ↑	[85]
	Nanoparticle	ES100	5-ASA	DSS, UC mice	Colon targeting, inflammation ↓, mucosal barrier ↑	[86]
	Nanoparticle	HA/PEI	RH	DSS, UC mice	Colon targeting, macrophage uptake ↑, inflammation ↓	[87]
	Nanoparticle	n/a	6-Shogaol + M2/M13	DSS, UC mice	Colon targeting, sustained release, inflammation ↓	[88]
	Liposome	CH/PT	Psoralen	DSS, UC mice	Colon targeting, inflammation ↓, oxidative stress ↓, mucosal barrier ↑	[89]
	Liposome	PT/TMC	Celastrol	DSS, UC mice	Colon retention ↑, inflammation ↓	[90]
	Mesoporous material	Azo-urea	Safranin O + HC	TNBS, UC mice	Colon targeting, inflammation ↓, retention ↑	[91]
	Hydrogel	n/a	5-ASA	UC mice	Colon targeting, retention ↑, colitis symptoms ↓	[92]
Regulate immune inflammation	Nanoparticle	FA	6-Shogaol	DSS, UC mice	Inflamed-colon targeting, uptake ↑, ulcer healing ↑	[75]
	Liposome	n/a	siCD98	UC mice	CD98 ↓, inflammation ↓	[76]
	Nanoparticle	n/a	Tpl	UC mice	Colon targeting, ROS ↓, inflammation ↓	[93]
	Nanoparticle	n/a	LMWH	UC mice	Tissue targeting, macrophage cytokines ↓, drug protection ↑	[94]
	Nanoparticle	ATP-CMI	BUD	Colitis mice	Lesion accumulation ↑, redox-triggered release	[95]
	Micelle	GC-boronate	Que	Colitis mice	Lesion accumulation ↑, pH/ROS-responsive release, inflammation ↓	[96]
	Liposome	n/a	SOD	DSS, UC mice	Oxidative stress ↓, inflammation ↓, barrier protection ↑	[97]
	Nanoparticle	n/a	Dex + butyrate	DSS, UC mice	Cell adhesion ↓, pro-inflammatory factors ↓, anti-inflammatory effect ↑	[98]
	Nanoparticle	Man	n/a	Colitis mice	Macrophage targeting ↑, imaging/therapy integration ↑	[99]
	Exosome	n/a	NAI	DSS, acute colitis mice	Pro-inflammatory factors ↓, anti-inflammatory factors ↑	[100]
	Exosome	EphB2	NAI	DSS, UC mice	Immune balance ↑, inflammation ↓	[101]
Restoring the Barrier and the Microbiome	Hydrogel	CMCHD/HP-β-CD	RNs + Kae	UC mice	Inflammation ↓, oxidative stress ↓, intestinal barrier ↑	[82]
	Exosome	n/a	NAI	DSS, colitis mice	Microbiota homeostasis ↑, T-cell response ↑	[83]
	Exosome	n/a	NAI	DSS, UC mice	Microbiota homeostasis ↑, SCFAs ↑, bile acid metabolism ↑	[84]
	Nanoparticle	n/a	Natural cargo	DSS, IBD mice	IEC survival/proliferation ↑, intestinal repair ↑, inflammation balance ↑	[102]
	Hydrogel	PAA	CeO_2_ NPs	IBD mice	Free radicals ↓, oxidative inflammation ↓	[103]
	Hydrogel	PCLGA-PEG-PCLGA	5-ASA + CUR	UC mice	Sustained release, inflammation ↓, mucosal barrier ↑	[104]
	Exosome	n/a	NAI	UC mice	Microbiota homeostasis ↑, tryptophan metabolism ↑, barrier protection ↑	[105]
	Exosome	n/a	NAI	DSS, colitis mice	NETs ↓, zinc homeostasis ↑	[106]
	Exosome	n/a	NAI	DSS, UC mice	Microbiota/tryptophan metabolism ↑, oxidative stress ↓, inflammation ↓	[107]
	Exosome	n/a	NAI	DSS, colitis mice	NLRP3 signaling ↓, microbiota homeostasis ↑	[108]
	Exosome	n/a	CX5461	DSS, colitis mice	Pro-inflammatory factors ↓, M2 polarization ↑	[109]
	Exosome	n/a	NAI	DSS, colitis mice	IEC protection ↑, colitis ↓	[110]
	Exosome	n/a	NAI	DSS, colitis mice	Microbiota homeostasis ↑, M2 polarization ↑	[111]

Table notes: n/a (not applicable or not available), ↑ (up-regulated, increase, activate), ↓ (down-regulated, decrease, inhibit), 5-ASA (5-aminosalicylic acid), BUD (budesonide), CeO_2_ NPs (cerium dioxide nanoparticles), CH (chitosan), CMCHD (carboxymethyl chitosan derivative), CUR (curcumin), Dex (dexamethasone), DSS (dextran sulfate sodium), HA (hyaluronic acid), HC (hydrocortisone), HP-β-CD (hydroxypropyl-β-cyclodextrin), IBD (inflammatory bowel disease), IEC (intestinal epithelial cell), Kae (kaempferol), LMWH (low-molecular-weight heparin), Man (mannosylated), NAI (naturally occurring active ingredients), NETs (neutrophil extracellular traps), PAA (polyacrylic acid), PEG (polyethylene glycol), PEI (polyethyleneimine), PT (pectin), Que (quercetin), RNs (rhubarb-derived nanobubbles), ROS (reactive oxygen species), SCFAs (short-chain fatty acids), siCD98 (small interfering RNA targeting CD98), SOD (superoxide dismutase), SSZ (sulfasalazine), TMC (trimethyl chitosan), TNBS (2,4,6-trinitrobenzenesulfonic acid), Tpl (tempol), UC (ulcerative colitis).

### 2.3. Applications of Nano-Drug Delivery Systems in RA

RA is a chronic autoimmune disease that primarily affects the joints and surrounding soft tissues. It is closely associated with progressive functional decline, premature mortality, and a significant socioeconomic burden [112]. Globally, the prevalence of RA varies widely, ranging from 0.25% to 1% [113], with women being approximately two to three times more likely to be affected than men [114]. RA is currently considered a multifactorial disease involving complex interactions between host-specific and environmental factors, which collectively influence susceptibility, disease persistence, and severity. Regarding host-related factors, the risk of developing RA can be attributed to genetic, epigenetic, reproductive, neuroendocrine mechanisms, and hormonal levels. Environmental risk factors include dietary patterns, smoking, the microbiome and infectious agents, other air pollutants, and socioeconomic conditions [115]. Current treatment approaches are primarily based on surgical and pharmacological interventions, including biologics, nonsteroidal anti-inflammatory drugs, disease-modifying antirheumatic drugs (DMARDs), glucocorticoids, and gene therapy [116]. Taking glucocorticoids as an example, although they possess significant immunomodulatory and anti-inflammatory effects and are widely used to treat RA, adverse reactions such as weight gain, peptic ulcers, and even psychiatric abnormalities have been observed in patients receiving glucocorticoid therapy [117]. Given the broad-spectrum anti-inflammatory effects of nanoparticles, they have been applied in the management of RA. Nanoparticles can neutralize pro-inflammatory cytokines, suppress synovial inflammation, penetrate deep into the cartilage matrix, and provide robust cartilage protection to prevent joint damage [118]. Nanoparticle delivery systems offer a precise and efficient strategy for RA treatment by targeting inflamed joints, modulating the immune response, and promoting cartilage repair. Applications of specific nano-drug delivery systems in RA are summarized in Table 3.

#### 2.3.1. Targeted Delivery to Joints

Increased macrophage infiltration and vascular permeability are major pathological features of RA, providing favorable conditions and target cells for nanomedicine delivery systems [119]. Wang et al. [120] developed a targeted exosome therapeutic system using exosomes derived from anti-inflammatory M2 macrophages as a platform, simultaneously modifying their membrane surface with poly-L-lysine and matrix metalloproteinase (MMP)-cleavable polyethylene glycol (PEG). Following intravenous injection, PEG prolongs circulation time, and in conjunction with chemokine ligand-mediated active targeting, achieves accumulation in inflamed joints, while the exosomes themselves induce macrophage polarization toward the M2 phenotype. Oral nanoparticle formulations offer unique advantages in terms of patient compliance and long-term safety. Folic acid (FA)-modified chitosan (CS)-coated SLNs were prepared using a layer-by-layer coating technique to load leflunomide. Following oral administration, these nanoparticles significantly improved joint healing, reduced hepatotoxicity, and enhanced anti-inflammatory effects through folate receptor-mediated targeting [121]. In addition to serving as drug delivery carriers, inorganic nanoparticles often possess intrinsic therapeutic functions (such as scavenging ROS). However, traditional inorganic nanomaterials are prone to being coated with a protein shell in vivo, thereby reducing their therapeutic efficacy. To address this issue, Jia et al. [122] loaded methotrexate (MTX) into hollow manganese dioxide nanoparticles (H-MnO_2_ NPs) and formed a “pseudo-protein corona” coating using HSA at physiological concentrations, yielding HSA-MnO_2_@MTX NPs. Compared with uncoated nanoparticles, the HSA coating more effectively reduced pro-inflammatory cytokine levels and inhibited the accumulation of ROS. HSA-MnO_2_@MTX NPs exhibited improved biodistribution, specifically targeted the ankle joint, modulated pro-inflammatory cytokine production, and limited cartilage degradation and signs of inflammation.

#### 2.3.2. Regulation of the Immune Response

The onset and progression of RA are closely associated with the abnormal activation of immune cells, such as macrophages and T cells, as well as inflammatory cytokines. Macrophages are key effector cells in the pathological process of RA. These cells can interact with T cells, B cells, and fibroblast-like synoviocytes, producing large amounts of cytokines, chemokines, digestive enzymes, and ROS, thereby accelerating bone destruction. Therefore, the use of nanomaterials to target macrophages for the treatment of RA is of critical importance [118]. Polymer micelles are core-shell structures formed by the self-assembly of amphiphilic block copolymer molecules. The inner core of polymer micelles consists of hydrophobic segments within the copolymer, enabling them to encapsulate poorly water-soluble drugs, antifungal agents [123], and polynucleotides [124]. Meanwhile, the hydrophilic segments form the outer layer of the micelle, enabling it to remain stable in biological systems [125]. The core-shell structure of polymer micelles allows for efficient drug loading and protection against interference from the aqueous environment, making them highly promising for applications in drug delivery. Yu et al. [126] prepared folic acid (FA)-modified micelles based on Panax notoginseng polysaccharide-deoxycholic acid (DC) conjugates for the targeted delivery of polyphyllin I (PPI) to macrophages, naming them FA-PPI-Ms. FA-PPI-Ms undergo FA receptor-mediated endocytosis, scavenge ROS, and inhibit the JAK2/STAT3 signaling pathway, promoting the polarization of macrophages from the M1 to the M2 phenotype, thereby significantly alleviating joint swelling and synovial inflammation. Dendritic macromolecules are a class of polymeric materials with precisely defined three-dimensional branched conformations. They have demonstrated significant application value in the fields of integrated neurodiagnosis and -therapy as well as biomedicine, and have attracted particular attention as nanoscale drug carriers [127]. The particle size, molecular weight, and solubility properties (including water solubility) of dendrimers can all be flexibly controlled, making them functional materials with an extremely broad range of applications. These macromolecules inherently possess cavity structures capable of accommodating guest molecules, and their surfaces can be engineered to incorporate various functional groups, such as targeting ligands and charged groups, thereby enhancing the material’s biocompatibility while reducing its own toxicity [128,129]. Another study utilized fluorinated polyamidine dendrimer (FP) to deliver miR-23b (FP/miR-23b). Following intravenous injection, FP/miR-23b nanoparticles preferentially accumulated in inflamed joints and were nonspecifically taken up by synovial cells, thereby restoring miR-23b expression in synovial tissue. miR-23b inhibits the NF-κB pathway, reduces pro-inflammatory factors, and induces apoptosis in activated macrophages. Importantly, FP/miR-23b did not cause significant systemic toxicity [130]. As endogenous nanocarriers, exosomes offer advantages such as low immunogenicity, high biocompatibility, and the ability to carry various bioactive molecules. Li et al. [131] utilized exosomes derived from M2 macrophages (M2-Exos) for the treatment of RA. In this study, plasmid DNA (pDNA) encoding the anti-inflammatory cytokine IL-10 was co-loaded into M2-Exos with the glucocorticoid betamethasone sodium phosphate, creating a biomimetic co-delivery system (M2 Exo/pDNA/BSP). M2 Exo/pDNA/BSP significantly suppressed the secretion of pro-inflammatory factors (IL-1β, TNF-α) and upregulated the expression of the anti-inflammatory factor IL-10, thereby effectively inducing the polarization of macrophages from the M1 phenotype to the M2 phenotype. Furthermore, exosome-targeted delivery allows for a reduction in the dosage of glucocorticoids, thereby lowering the risk of side effects associated with conventional administration methods. Consequently, M2 exosomes, as a biomimetic nanoplatform, offer a new strategy for the immunotherapy of RA by reprogramming the polarization state of macrophages.

#### 2.3.3. Promoting Cartilage Repair

Advanced RA is often accompanied by irreversible cartilage and bone erosion. The low-cell-density characteristics and acellular nature of cartilage tissue make cartilage repair extremely difficult. Promoting cartilage repair is key to improving prognosis, and nanocarriers offer new opportunities for effective cartilage repair [132]. Nanocarriers can achieve local drug delivery via transdermal routes, thereby enhancing bioavailability. Simultaneously, by utilizing biomimetic microneedle structures to penetrate the skin’s stratum corneum barrier, they precisely deliver therapeutic drugs to the joint cavity, thereby inhibiting the inflammatory death of synovial cells and creating a favorable microenvironment for cartilage repair. Zheng et al. [133] designed and fabricated a biomimetic, double-crosslinked hydrogel-based soluble microneedle (MN). This MN, based on GelMA and methacrylated fibrinogen, is used for transdermal delivery of quercetin liposome nanoparticles modified with the transmembrane peptide TAT (QUE-Lipo-TAT). QUE-Lipo-TAT can block the pyroptosis pathway in RA-FLS by specifically inhibiting caspase-8, thereby preventing pyroptosis in RA-FLS while effectively suppressing inflammatory cell death, abnormal cell proliferation, and migration, thus providing the prerequisites for cartilage self-repair. Regulating the polarization state of macrophages can also alleviate joint inflammation, thereby protecting cartilage from erosion. Exosomes (ADSCs-EXO) were extracted from adipose-derived stem cells and loaded with icariin (ICA) to construct the ADSCs-EXO-ICA delivery system. ADSCs-EXO-ICA can reduce glycolysis levels by inhibiting the ERK/HIF-1α/GLUT1 signaling pathway. Furthermore, ADSCs-EXO-ICA effectively accumulates at joint sites, reducing levels of inflammatory cytokines, alleviating synovitis, and protecting cartilage integrity [134]. Regulating the gut microbiota-immune axis also offers a new approach to cartilage protection in RA. Han et al. [135] isolated and extracted ELNs from Pueraria root(Pu-ELNs)to target *Ruminococcus gnavus*, a pathogenic bacterium enriched in the intestines of RA patients. Mechanistic studies have shown that *R. gnavus* induces excessive neutrophil extracellular trap (NET) formation by secreting phenylethylamine (PEA), thereby exacerbating arthritis. Pu-ELNs can inhibit the expression of phenylalanine decarboxylase, reduce PEA production, and thereby alleviate arthritis symptoms. By regulating gut microbiota metabolism, Pu-ELNs indirectly alleviate joint inflammation and cartilage destruction.

**Table 3:** Applications of nano-drug delivery systems in the treatment of RA.

Therapeutic Strategies	Types of Nanocarriers	Surface Finishing	Drug-Loaded	Animal Models	Function	References
Targeted delivery to joints	Exosome	Oligolysine + MMP-cleavable PEG	Exosomes + cfDNA scavenging	CIA mice	Joint targeting, cfDNA ↓, M2 polarization ↑	[120]
	Nanoparticle	CS/FA	Lef	AIA rats	FR targeting, sustained release, joint repair ↑	[121]
	Nanoparticle	HSA	MTX	CIA rats	Ankle targeting, ROS/inflammation ↓, synoviocyte proliferation ↓	[122]
	Nanoparticle	MSC membrane (LFA-1/ICAM-1)	Dex	CIA mice	Joint targeting, inflammation ↓, cartilage protection ↑	[136]
	Liposome	CK	Dex	CIA mice	Joint targeting, anti-inflammation ↑	[137]
	Liposome	HA	DSP nanogel	CIA rats	Joint targeting, inflammation ↓, cartilage repair ↑	[138]
	Liposome	PDA	MTX + O2 generator	AIA rats	Joint targeting, hypoxia/ROS ↓, synergistic therapy ↑	[139]
	Nanoparticle	HA + mixed membranes	IND	AIA rats	Joint targeting, pH-responsive release, inflammation ↓	[140]
	Exosome	CD90 Ab	PB	CIA mice	Inflammation ↓, joint swelling ↓	[141]
Regulate the immune response	Micelle	FA	PPI	CIA rats	JAK2/STAT3 ↓, M2 polarization ↑	[126]
	Dendrimer	FP	miR-23b	AIA rats	Macrophage apoptosis ↑, NF-kB signaling ↓	[130]
	Exosome	n/a	IL-10 pDNA + BSP	CIA mice	M1-to M2 reprogramming ↑, inflammation ↓	[131]
	Hydrogel	n/a	SIN + GA	AIA mice	Neutrophil overactivation ↓, apoptosis normalization ↑	[142]
	Mesoporous material	n/a	Zn-Cur	AIA mice	Antioxidant activity ↑, M2 polarization ↑, mineralization ↑	[143]
	Nanoparticle	PCL-AC	GA + Bud	CIA rats	Inflammation ↓, bone/cartilage damage ↓, joint histology ↑	[144]
	Nanoparticle	CD44/FR ligand	RBA	AIA rats	Targeted delivery, ERK/HIF-1alpha/GLUT1 ↓	[145]
	Nanoparticle	n/a	miR-124 + Ket	AIA rats	Acid-responsive release, inflammation ↓, arthritis progression ↓	[146]
	Nanoparticle	RGD + MMP-9-cleavable PEG	CEL	AIA rats	Macrophage/osteoclast targeting, apoptosis ↑	[147]
	Nanoparticle	n/a	IL-10 pDNA + DSP	CIA rats	Synovial macrophage targeting, M1 to M2 polarization ↑	[148]
	Micelle	n/a	TP + VP	CIA mice	Inflammation/oxidative stress ↓, swelling/bone erosion ↓	[149]
	Dendrimer	FP	miR-30a	CIA mice	NF-kB/MAPK ↓, arthritis ↓	[150]
	Dendrimer	n/a	n/a	CIA rats	cfDNA ↓, joint inflammation ↓	[151]
	Hydrogel	n/a	DNase I	CIA mice	Inflammatory factors ↓, arthritis symptoms ↓	[152]
	Exosome	M2 exosome membrane	CuS + CitP + RapA	CIA mice	T-cell apoptosis ↑, immune tolerance ↑	[153]
	Exosome	n/a	IkBalpha inhibitor	SKG and CIA mice	NF-kB ↓, inflammation ↓, cartilage damage ↓	[154]
	Exosome	FA	GDEV cargo	CIA mice	M1 macrophage targeting, PI3K-AKT modulation	[155]
	Exosome	n/a	PD-L1 cargo	CIA mice	Joint targeting, T-cell activity ↓	[156]
	Nanoparticle	n/a	NAI	CIA mice	anti-inflammatory, MAPK ↓	[157]
	Exosome	n/a	NAI	CAIA mice	attenuated synovitis, arthritis severity ↓	[158]
	Exosome	n/a	CTLA-4Ig cargo	CIA mice	Immune modulation ↑, cartilage protection ↑	[159]
Promotes cartilage repair	Hydrogel	TAT (DSPE-PEG2K-TAT)	Que	CIA rats	Pyroptosis/necroptosis ↓, inflammation/synovial hyperplasia ↓	[133]
	Exosome	n/a	ICA	CIA rats	M1 to M2 polarization ↑, synovitis ↓, cartilage protection ↑	[134]
	Exosome	n/a	gma-miR4412	CIA mice	NETs ↓, arthritis ↓, cartilage protection ↑	[135]
	Hydrogel	n/a	MTX NP + PEITC NE	AIA rats	Anti-inflammation/chondroprotection ↑, cartilage degradation ↓	[160]
	Hydrogel	n/a	MTX	AIA rats	Cartilage repair ↑, hyperalgesia ↓	[161]
	Exosome	n/a	SiO_2_-MTX	AIA and CIA mice	M2 polarization ↑, cartilage protection ↑	[162]
	Exosome	LMWH (ROS-responsive linker)	Dex	CIA mice	Neutrophil apoptosis ↑, oxidative damage ↓	[163]

Table notes: n/a (not applicable or not available), ↑ (up-regulated, increase, activate), ↓ (down-regulated, decrease, inhibit), AIA (adjuvant-induced arthritis), BSP (betamethasone sodium phosphate), cfDNA (cell-free DNA), CEL (celastrol), CIA (collagen-induced arthritis), CAIA (collagen antibody-induced arthritis), CitP (citrullinated peptide), CS (chitosan), CTLA-4Ig (cytotoxic T-lymphocyte-associated protein 4 immunoglobulin), CuS (copper sulfide), Dex (dexamethasone), DNase (deoxyribonuclease), DSP (sodium dexamethasone phosphate), FA (folic acid), FP (fluorinated polyamidine dendrimer), FR (folate receptor), GA (glycyrrhizic acid), GDEV (ginger-derived extracellular vesicle), HA (hyaluronic acid), HSA (human serum albumin), ICA (icariin), ICAM-1 (intercellular adhesion molecule-1), IkBalpha (inhibitor of kappa B alpha), IL-10 pDNA (plasmid DNA encoding interleukin-10), IND (indomethacin), LFA-1 (lymphocyte function-associated antigen-1), LMWH (low-molecular-weight heparin), MAPK (mitogen-activated protein kinase), MSC (mesenchymal stem cell), MTX (methotrexate), NAI (naturally occurring active ingredients), NETs (neutrophil extracellular traps), NP (nanoparticle), PD-L1 (programmed death-ligand 1), PEG (polyethylene glycol), PEITC (phenethyl isothiocyanate), PPI (polyphyllin I), PB (Prussian blue), Que (quercetin), RBA (roburic acid), RGD (arginine-glycine-aspartic acid), ROS (reactive oxygen species), SiO_2_ (silica), SIN (sinapine), SKG (SKG arthritis model), TAT (trans-activator of transcription peptide), TP (trichosanthin), VP (L-ascorbyl palmitate), Zn-Cur (zinc-curcumin).

## 3. Conclusions and Future Prospects

Nano-drug delivery systems have opened up new avenues for the treatment of chronic autoimmune diseases, including DM, IBD, and RA. By enabling targeted delivery, controlled release, and improved pharmacokinetic behavior, nanotechnology has significantly overcome the limitations of traditional immunosuppressive therapies, such as low bioavailability, severe off-target effects, and poor patient compliance. Various nanocarriers, such as polymeric nanoparticles, liposomes, micelles, dendrimers, mesoporous silica, hydrogels, and exosomes, have demonstrated promising results in preclinical studies, capable of modulating immune responses, reducing inflammation, and promoting tissue repair.

Despite these advances, the clinical translation of nanomedicines in the field of autoimmune diseases still faces significant challenges. Currently, most studies remain in the preclinical stage, and there is an urgent need to establish standardized protocols for large-scale production and a comprehensive toxicological evaluation system. Thus, future studies must involve the development of personalized nanocarriers specific to individual patient immune profiles. They must also include engineering intelligent, responsive delivery systems, like carriers responsive to ROS, pH, or enzymes, to facilitate on-demand drug delivery depending upon disease activity. Diagnostics-therapeutics integration, that is, combining diagnostic and therapeutic activities to observe the response to the treatment in real time, is another key direction. Furthermore, investigating noninvasive administration routes, including oral, transdermal MN, and inhalation, is necessary to enhance the compliance with long-term treatment. Before regulatory acceptance, the risk-benefit analysis of the long-term organ toxicity of nanomedicines, such as effects on the liver, kidneys, spleen, and lungs, as well as their possible immunosuppressive properties, is required to be performed carefully. With the deepening of multidisciplinary collaboration, nanotechnology is expected to drive a fundamental shift in the management of chronic autoimmune diseases, moving from symptom control toward immune modulation and ultimately the restoration of immune tolerance.

## Data Availability

No new data were created or analyzed in this study. Data sharing is not applicable.

## Abbreviations

The following abbreviations are used in this manuscript:

BSP	Betamethasone sodium phosphate
Cat-CS	Catechol-modified chitosan
CD	Crohn’s disease
CUR-LPs	Curcumin-loaded anionic liposomes
DC	Deoxycholic acid
DMARDs	Disease-modifying antirheumatic drugs
DR	Diabetic retinopathy
FN	Fibronectin
FP	Fluorinated polyamidine dendrimer
GA	Glycyrrhetinic acid
GelMA	Gelatin Methacryloyl
GDLVs	Ginger-derived lipid vesicles
HNVs	Honeysuckle-derived nanovesicles
HSA	Human serum albumin
IBD	Inflammatory bowel disease
ICA	Icariin
Kae	Kaempferol
MMP	Matrix metalloproteinase
MSNs	Mesoporous silica nanoparticles
M2-Exos	Exosomes derived from M2 macrophages
MN	Microneedle
MTX	Methotrexate
pDNA	plasmid DNA
PEA	Phenylethylamine
PEG	Polyethylene glycol
PELNs	Exosome-like nanoparticles isolated from edible Portulaca oleracea L
PPI	Polyphyllin I
Pu-ELNs	Pueraria lobata-derived exosome-like nanovesicles
RA	Rheumatoid arthritis
RNs	Rhubarb-derived nanovesicles
ROS	Reactive oxygen species
SLNs	Solid lipid nanoparticles
SSZ	Sulfasalazine
UC	Ulcerative colitis
